# Unveiling the Potential of Natural Deep Eutectic Solvents in Electrochemical Energy Storage Applications

**DOI:** 10.1021/acsphyschemau.5c00063

**Published:** 2025-08-15

**Authors:** Henrique de Araujo Chagas, Guilherme Colherinhas, Eudes E. Fileti

**Affiliations:** † Instituto de Física, 67824 Universidade Federal de Goiás, Goiânia, Goiás 74690-900, Brazil; ‡ Instituto de Ciência e Tecnologia, Universidade Federal de São Paulo, São José dos Campos, São Paulo 12247-014, Brazil

**Keywords:** eutectic solvents, NADES electrolyte, eupercapacitor, graphene, molecular dynamics

## Abstract

Supercapacitors are key to sustainable energy storage due to their high power density and long lifespan, though their energy density remains limited. This study explores natural deep eutectic solvents (NADES) as alternative electrolytes for graphene-based supercapacitors via molecular dynamics simulations. Three NADES—composed of betaine chloride and the amino acids arginine, histidine, or lysine—are assessed for their biocompatibility, cost-effectiveness, and hydrogen-bonding capabilities. Simulations at 300 and 600 K reveal distinct physicochemical behaviors: histidine-based NADES shows the highest density and cohesive energy, attributed to imidazole-mediated interactions, while lysine-based NADES offers the greatest ionic mobility. In supercapacitor models, asymmetric electric double layers (EDLs) form, with amino acids dominating the positive EDL and betaine the negative. Interaction energy analyses underscore the stabilizing role of amino acids in the EDL structure. Capacitance values range from 2.2 to 2.8 μF/cm^2^, aligning with those of conventional electrolytes. These results highlight the promise of NADES as sustainable and tunable electrolytes, offering a viable route to enhance the performance of next-generation supercapacitors.

## Graphical abstract

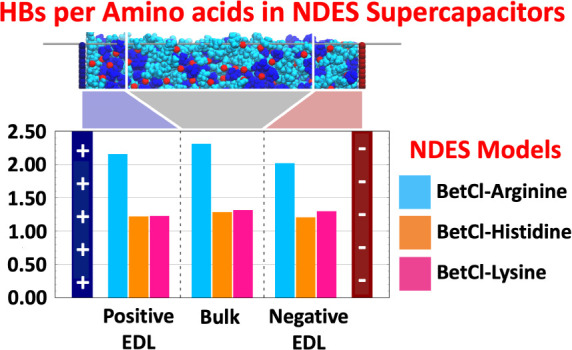

## 1 Introduction

Electrochemical energy storage devices play a crucial role in the transition to sustainable energy systems.^1–3^ Among these devices, supercapacitors, also known as ultracapacitors, stand out for their high power density and long lifespan, although they have limitations in energy density, which restricts their use in high-energy-demand applications.^1–3^ To overcome this challenge, advances in the composition and performance of electrolytes have received increasing attention, as the energy density of electrochemical supercapacitors is directly related to the capacitance and the stable potential window of the electrolyte.^3^

Currently, various electrolyte alternatives are being explored.^3^ Aqueous electrolytes have high ionic conductivity but are limited by a narrow potential window due to water decomposition.^4^ Organic electrolytes offer greater electrochemical stability but face challenges related to toxicity, flammability, and purification complexity.^5^ Ionic liquids, in turn, have demonstrated excellent thermal stability and a wide potential window but have limitations in cost and high viscosity, hindering their large-scale application.^6^

Recently, ionic liquids (ILs) and deep eutectic solvents (DES) have emerged as promising alternatives to overcome these limitations.^7,8^ While ionic liquids, for example, stand out for their wide electrochemical stability window and high conductivity, their high cost and viscosity still restrict their practical applicability.^6^ DES, on the other hand, provide a lower-cost solution, greater synthesis simplicity, and adjustable properties that make them particularly attractive. These solvents have a unique combination of characteristics, such as thermal stability, low toxicity, and excellent ionic behavior, especially at elevated temperatures.^7,8^ Their formation is based on strong interactions between metal salts and compounds with high electron-donating ability, resulting in electrochemical properties that can be optimized for supercapacitor applications. In this context, natural deep eutectic solvents (NADES) are further highlighted, as they combine the typical properties of DES with other desirable attributes, such as biodegradability, low toxicity, and ease of synthesis.^8–10^ NADES have shown great potential as solvents or supporting media in various processes, ranging from DNA solubilization,^11^ enzymatic reactions,^12^ active pharmaceutical ingredients,^13^ CO_2_ capture,^14^ to electrodeposition applications.^15^ However, their use as electrolytes or additives in energy storage devices, such as supercapacitors, remains unexplored, representing a promising opportunity for future investigations.

In this work, we will conduct a series of classical molecular dynamics simulations to explore the potential of NADES as electrolytes for supercapacitors. Our goal is to investigate not only the intrinsic properties of these NADES as pure electrolytes but also their interaction in energy storage devices based on graphene electrodes. We will focus on NADES formed from betaine/chloride as hydrogen bond acceptors (HBA) and amino acids such as arginine, histidine, and lysine as hydrogen bond donors (HBD). These amino acids were selected due to their promising characteristics, such as low toxicity, low cost, and the ability to form specific interactions via hydrogen bonds. By employing three different NADES with adjustable properties, we aim to advance the understanding of their applicability as electrolytes in graphene supercapacitors, contributing to the development of more efficient and sustainable energy storage systems.

## 2 Methods

Classical molecular dynamics (MD) simulations were performed to determine the structural and electrostatic properties of three betaine-amino acid–based natural deep eutectic solvents (Bet-AA DES):^16^ BetCl-Arginine, BetCl-Histidine, and BetCl-Lysine, used as electrolytes (with BetCl and each amino acid species mixed in a 1:1 molar ratio) in graphene-based supercapacitors. Two series of simulations were carried out: the first to obtain the physical properties of the pure electrolytes at two temperatures, 300 and 600 K, and another series of simulations aimed at studying the structural and electrostatic properties of electrolyte cells. The first series consists in simulations of cubic boxes with dimensions of 4 × 4 × 4 nm^3^ for each one of the three electrolytes. With the PACKMOL package,^17^ 137, 144, and 135 pairs of BetCl-Arg, BetCl-His and BetCl-Lys, respectively, were randomly distributed in these boxes. In these systems, temperatures of 300 and 600 K were maintained with v-rescale^18^ technique with time constant of 0.1 ps. The reference pressure of 1.013 bar was controlled via the Parrinello–Rahman barostat^19^ with time constant of 0.5 ps under the isothermal–isobaric (NPT) ensemble. Electrostatic interactions were computed using the Particle-Mesh Ewald (PME) method^20^ with a cutoff radius of 1.2 nm, while van der Waals interactions were modeled using the cutoff scheme with a Verlet modifier and the same radius of 1.2 nm. After reach the thermodynamic equilibrium, a production run of 30 ns was performed to calculate the density of these liquids, which would subsequently be used to fill the supercapacitors in the second series of simulations. For this second series, simulation boxes were constructed using PACKMOL,^17^ with species randomly distributed to match the desired density, which was adjusted by properly setting the *z*-axis distance between the electrodes. The dimensions of the graphene electrodes were 3.71 × 3.85 nm^2^, and they were separated by approximately 12 nm, within which the electrolytes were inserted. A 24 nm vacuum slab was added between the electrodes to prevent spurious interactions. The interelectrode distance was set to 12 nm for each system. Two-dimensional periodic boundary conditions were applied in the xy-plane. A molecular representation of each electrolyte components and each electrolyte cell is shown in Figure 1.

**Figure 1:**
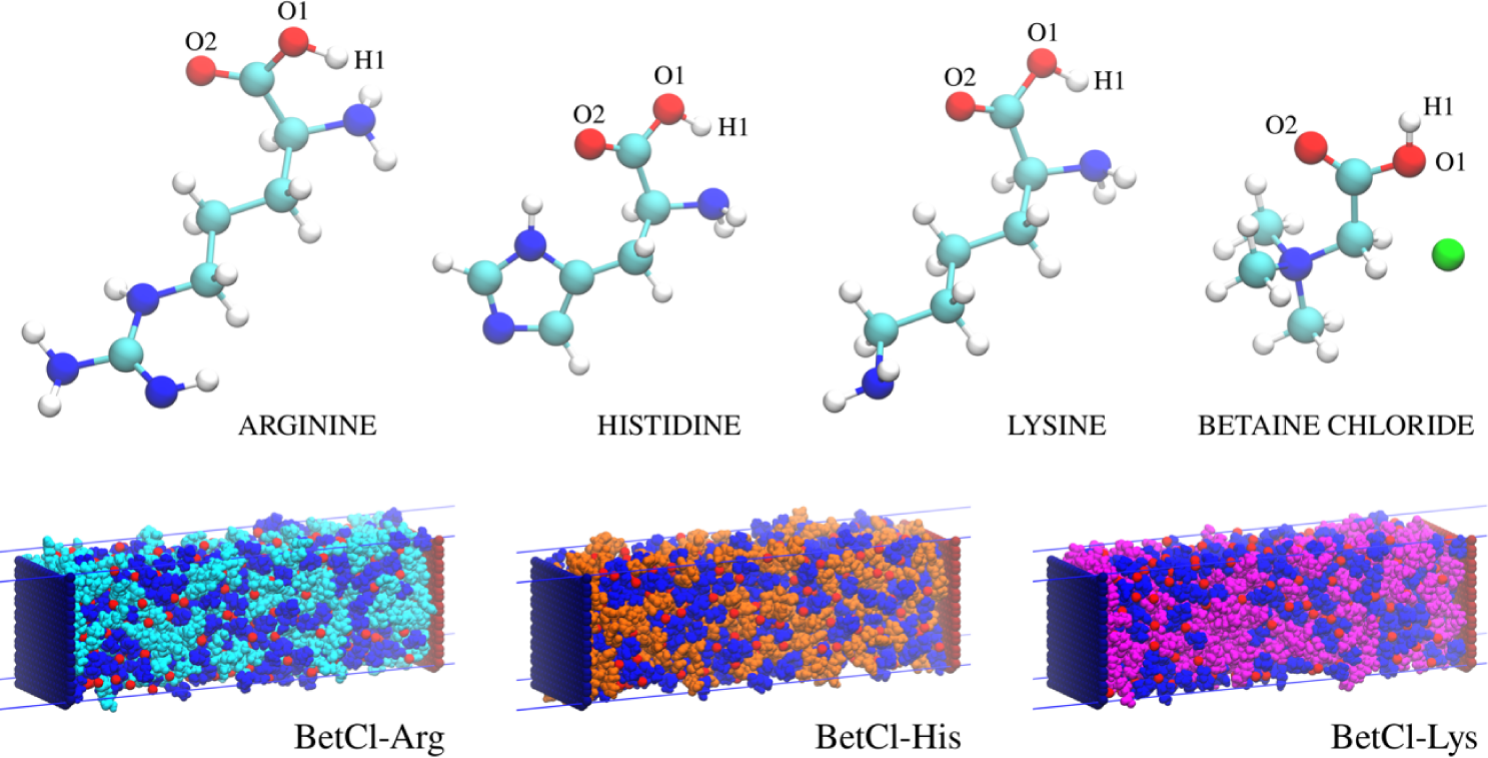
Molecular structures of each electrolyte component and each BetCl-AA DES as electrolytes in graphene-based supercapacitors. The supercapacitor model includes graphene electrodes, with the left electrode positively charged (blue) and the right electrode negatively charged (red).

The systems were pre-equilibrated for 15 ns in the canonical isothermal-isovolumetric (NVT) ensemble with a time step of 0.001 ps, followed by a 30 ns production run. Since the Bet-AA DES are highly viscous, all systems were simulated at a temperature of 600 K, intentionally higher than the critical temperature to accelerate dynamics and prevent the formation of multiple metastable states, which are common in many ILs at room temperature. This elevated temperature was not intended to reflect the operating conditions of real devices, but rather to enhance sampling efficiency and probe the stability of intermolecular interactions. Importantly, structural properties such as density profiles, hydrogen-bond networks, and interfacial organization tend to vary modestly with temperature, especially above the glass transition but below decomposition thresholds. Thus, while quantitative transport properties (e.g., diffusion) are strongly temperature-dependent, the qualitative structural features central to this stud, such as EDL formation, energetic decomposition, and hydrogen-bond topology, are largely transferable to lower temperatures. Temperature and pressure coupling were applied via the Nose-Hoover and Parrinello–Rahman schemes, respectively.^19,21^ Electrostatic interactions were calculated using Coulomb’s law up to 1.2 nm, with the Particle-Mesh Ewald (PME) method employed beyond this range.^20^ Lennard-Jones interactions were tapered from 0 to 1.2 nm using the shifted force technique.^22^

The force field describing each system was adapted from All-Atom Optimized Potentials for Liquid Simulations (OPLS-AA) models,^23^ and the atomic charges on the ions were derived from CHELPG (Charges from Electrostatic Potentials using a Grid-based method)^24^ quantum mechanical calculations at the 6–311++G­(d,p)^25^ level using the Gaussian 16 program.^26^ The modeling of the electrolytes was carried out using a point charge model scaled by a factor of 0.8 (all files describing the system modeling are available in the Support Material). This is a standard procedure aimed at providing a more accurate representation of the system’s dynamic characteristics and has been successfully employed in numerous prior studies.^27^ For each model, four simulations were performed with different charge densities (σ) applied directly to the electrodes: 0.00, 1.60, 3.20, and 4.81 μC cm^–2^. This charge density was uniformly distributed across the atoms constituting the positive and negative graphene plates. The simulations were performed below the critical potential of ∼ 2 V. This is a well-established threshold above which significant field-induced modifications in the electronic structure of the electrode may occur. Staying within this limit ensures that electrostatic interactions between the electrolyte and the electrode remain in a linear physically realistic regime. Furthermore, we employed a fixed charge model, where partial charges are statically distributed over electrode atoms. This approach has been shown to yield consistent results in this voltage range, as it avoids artificial polarization effects that can arise beyond ∼ 2 V. Going above this threshold could introduce nonlinearities and compromise the reliability of fixed-charge models, particularly in terms of interfacial charge screening and ionic layering. The constant charge model yields excellent results for systems composed of graphene, simulated below the material’s critical potential (∼2 V), allowing for feasible behavioral trends compared to experimental results, at a significantly lower computational cost than methods such as constant potential applied to the electrodes. The LINCS (Linear Constraint Solver) algorithm was used to maintain the integrity of the molecules.^28^ All molecular dynamics calculations were performed using the GROMACS (Groningen Machine for Chemical Simulation) software.^29^

## 3 Results and Discussion

### Pure Electrolytes

DES often exhibit low ionic diffusivity, which is typically associated with poor electrolytic quality. However, this idea has recently been challenged by studies suggesting that such a characteristic, associated with high viscosity, not only does not negatively impact the electrolyte’s performance but may even be desirable. In this context, the electrolytes investigated here, despite exhibiting an intense network of electrostatic interactions as well as very slow dynamics (i.e., their diffusion coefficients are very low) at room temperature, display all the characteristics required to become excellent electrolytic solvents.

Figure 2 shows the physical properties of the investigated electrolytes at elevated temperatures (600 K). Figure 2a,b display the mass density and cohesive energy density. Note that these two properties are correlated: the denser the electrolyte, the higher the cohesive energy density. This makes sense since both properties are direct consequences of intermolecular interactions and indicate how strongly the electrolyte components interact with each other. The histidine-based NADES exhibits the highest density and cohesive energy. This NADES, among the three amino acids, is the only one containing an imidazolium ring. This ring can act as a proton donor or acceptor, promoting intermolecular interactions, facilitating hydrogen bonding and π-π interactions with other molecules, and thus leading to more efficient packing and consequently higher mass and cohesive energy density.

**Figure 2:**
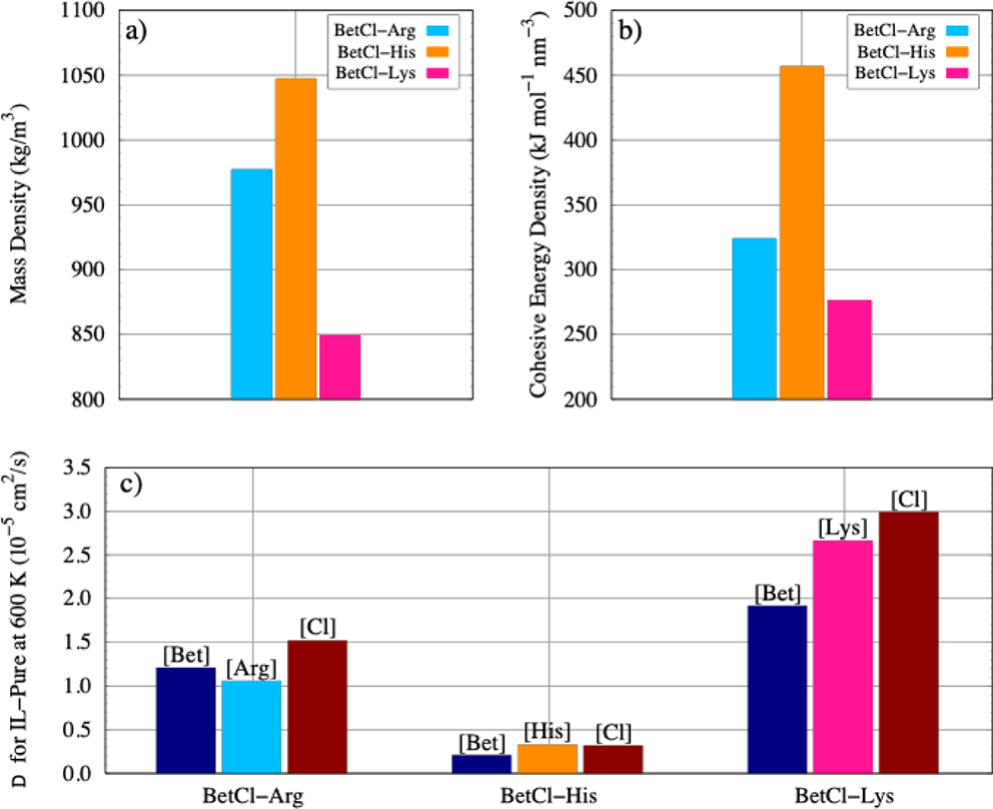
Physical properties of pure electrolytes at 600 K. Mass density (in kg cm^– 3^), cohesive energy density (kJ mol^– 1^ nm^– 3^), and diffusion coefficient (in cm^2^ s^– 1^). In plots a and b, the bars represent each of the electrolytes, while in plot c, the bars represent each component of a given electrolyte.

Figure 2c shows the diffusion coefficient for each component of the electrolytes. These values confirm the high sensitivity of ionic mobility to temperature and indicate that, on average, the diffusion coefficient at 600 K is approximately 15,000 times higher than at room temperature (values of D at 300 K are shown in the SI). This value is directly related to the viscosity of the electrolytes, which, although not determined in this study, can be inferred to be much lower at high temperatures. As expected, among the three DESs, the one based on lysine exhibited the highest diffusion coefficient, consistent with the lower intermolecular interaction observed for this system.

### Supercapacitors

A supercapacitor stores electrochemical energy through the rearrangement of electrolyte components near the electrified electrode surface during the formation of its electric double layer (EDL). In this context, describing the structural configuration of molecules within the EDL is crucial for understanding and characterizing the supercapacitor. One of the most insightful approaches to examining the local organization of ions on the electrode surface is through the analysis of the mass distribution profile. Figure 3 illustrates the density profile for each electrolyte investigated, highlighting both the positive and negative EDLs adjacent to the positive and negative electrodes, respectively.

**Figure 3:**
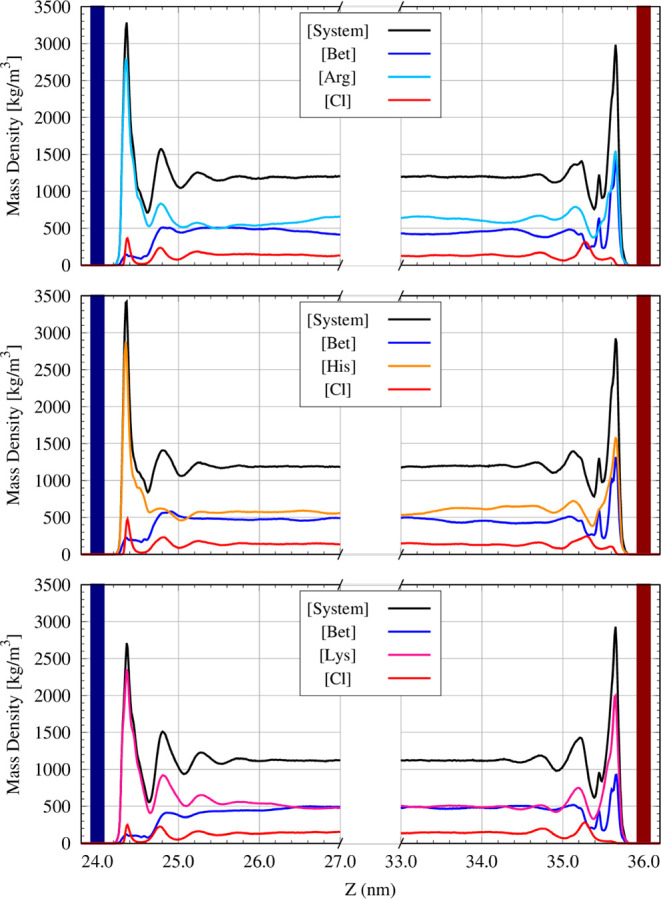
Mass density profile (in kg m^–3^) of the supercapacitors illustrating the electric double layer (EDL) near the positive (blue) and negative (red) electrodes. The results were derived for the electrodes with a surface charge density of 4.8 μC/cm^2^.

In all three cases, at the positive electrode, the EDL is characterized by the prominent presence of the amino acid, which governs the electrolyte distribution in this region (this can be observed by comparing the black curve, representing the overall electrolyte, with the colored curves corresponding to the amino acids). Additionally, a clear coordination between the amino acids (Arg, Lys, and His) and the chloride anion is evident. Here, the electrostatic interaction between the chloride anion and the positively charged electrode surface, as well as the strongly positive sites of the amino acids, is highly favored. Consequently, the distribution peaks for these two species are aligned at the positive electrode. On the other hand, at the negative electrode, while the electrostatic interaction between the electrode and the amino acids remains strong, the participation of betaine in the negative EDL is significant, shaping the overall electrolyte profile in this region.

Relevant information about the formation of the electric double layer can be obtained by analyzing the electrode–electrolyte interactions, which involve each component of the electrolyte and its dual interfaces with the electrodes, both positive and negative. These interactions, due to their range, predominantly occur near the electrodes and can characterize the energetics of the electric double layer. Figure 4 presents these data in a structured format as follows: the graph contains two columns, each representing the interaction with one electrode (positive and negative), and three rows, corresponding to energy types: Coulomb at the top, Lennard-Jones (LJ) in the middle, and total energy (the sum of Coulomb and LJ) at the bottom. All these energies are normalized relative to the number of Bet-Cl/AA pair into the boxes. Each plot in the figure displays three data blocks, one for each electrolyte component (Bet, AA, and Cl) and within each block, three columns specify the liquid to which the components pertain: BetCl-Arg (gray bars), BetCl-Hist (blue bars), and BetCl-Lys (red bars).

**Figure 4:**
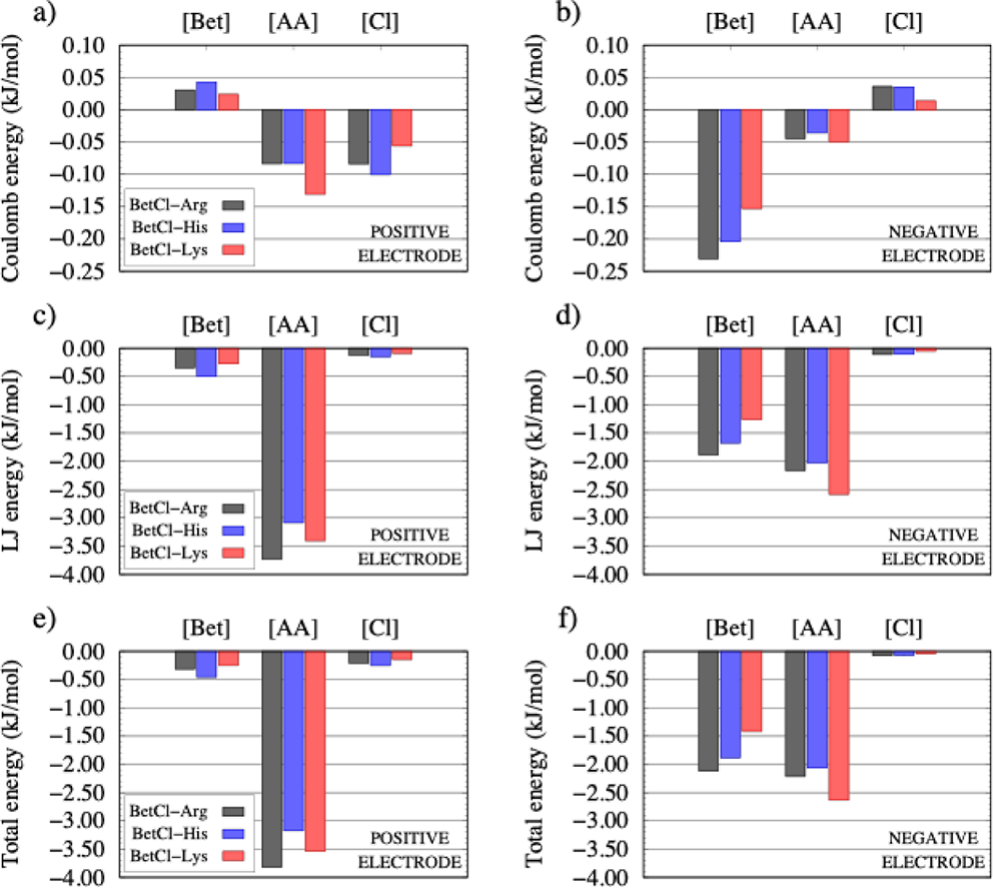
Decomposition of total pairwise interaction energies (in kJ mol^– 1^) into the Coulomb and Lennard-Jones (LJ) contributions. Energies are normalized per BetCl-AA ion pair. The statistical error is estimated to be within 1% for all reported values.

Overall, the interactions between the electrode and the electrolyte are predominantly governed by van der Waals forces, which are much stronger than electrostatic interactions. This occurs because Lennard-Jones (LJ) interactions of all species (positive or negative) with the electrodes are attractive. In contrast, Coulombic interactions can be either attractive or repulsive, depending on the net charge of the ion, leading to partial cancellation or even a repulsive force. For example, in the case of betaine, the Coulomb (Figures 4a,b) and LJ (Figures 4c,d) energies indicate that the interaction with the negative electrode is stronger than with the positive electrode. Furthermore, the LJ component is dominant, being up to 10 times greater than the Coulomb component. Notably, the Coulomb interaction of betaine with the positive electrode can be repulsive. This weaker interaction of betaine with the positive electrode (both Coulomb and LJ) is associated with the stronger interaction of the amino acid in the three liquids investigated (Figure 4a,c), suggesting that the electrolyte preferentially interacts with the positive electrode through amino acids. On the negative electrode, betaine interacts more strongly (Figure 4b) or similarly (Figure 4d) compared to the amino acid interaction. The total energy shows that, on the positive electrode, amino acids dominate the interaction (Figure 4e), while on the negative electrode, betaine and amino acids contribute comparably (Figure 4f). This asymmetry reflects the mass density profiles and may correlate with differences in the electrostatic profiles near the electrodes.

NADES stand out for their ability to form extensive hydrogen-bond networks, which directly impact critical properties such as viscosity, ionic mobility, and dissolution capacity.^9^ These networks increase viscosity and limit the mobility of ionic species, but the addition of water can flexibilization this structure, enhancing ionic conductivity. This ability also improves interactions with electrodes, promoting the formation of an efficient electric double layer, essential for high capacitance in supercapacitors.^2^ Furthermore, recent studies show that, although the high viscosity of electrolytes may reduce ionic mobility and hinder the formation of an effective electric double layer, it is not necessarily the limiting factor in the performance of electrochemical devices.^30^ Strategies such as the use of specific additives, which adjust viscosity or enhance electrode interactions, can mitigate these effects.^30^ In this context, NADES demonstrate significant potential, especially when combined with protective agents that optimize wettability and penetration into micropores, maximizing the use of the active surface and increasing device efficiency. Here, we present a detailed analysis of hydrogen bond (HB) formation in pure electrolytes and their interactions when confined in electrochemical cells bounded by graphene sheets. For supercapacitors, the analysis considered three distinct regions of the electrolyte: one near the positive electrode (EDL (+), 1 nm thick), another near the negative electrode (EDL(−), also 1 nm thick), and a central region (Bulk, 10 nm thick), as illustrated in Figure 5. Across the three electrolytes studied, no statistically significant differences were observed in the average number of HBs formed in these regions. This indicates that the hydrogen bond network within the electric double layers (EDLs) is essentially the same as in the bulk electrolyte. This uniformity, which is also linked to the viscosity of the electrolyte, has been observed in hydrated amino acid–based ionic liquids (ILAAs).^31^ A difference in water–water HB count was noted only when these amino acid–based ionic liquids were highly hydrated (∼90%). Under such conditions, the bulk region exhibited approximately 50% more HBs compared to the EDL regions. Thus, NADES in this context resemble ILAAs with low hydration levels. Despite the uniformity in the average HB count within the electrolyte in the device, distinct behaviors were observed for each amino acid studied. NADES based on arginine exhibited 2.0–2.5 HBs per amino acid within the device, decreasing to approximately 1.5 HBs per amino acid in the isolated liquid at 600 K. This demonstrates how confinement and the structural organization imposed by charged regions influence the system. Arginine shows about 1 HB/amino acid more than histidine and lysine in the electrolyte’s structural composition. Histidine and lysine showed approximately 1.0–1.3 HBs per amino acid within the device, about 50% higher than the 0.5–1.0 HBs per amino acid observed outside the device. It is also noteworthy that, despite the similar average HB counts in each region of the supercapacitor, the EDL (+) and EDL(−) compositions differ slightly in the number of amino acids. The EDL­(+) is more populated, with approximately 33, 35, and 41 amino acids for ILs composed of arginine, histidine, and lysine, respectively, while the EDL(−) contains approximately 30, 32, and 35 amino acids, respectively. In conclusion, while the average number of HBs in the EDLs is similar, the HB density per volume is higher near the positive electrode. This highlights a greater affinity of the amino acids for this region/electrode, corroborating the results shown in Figure 4.

**Figure 5:**
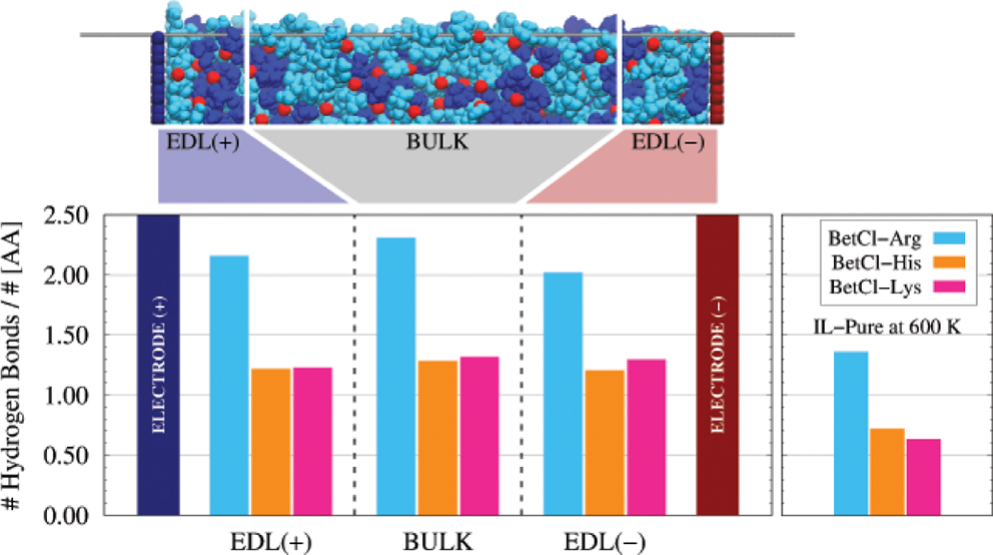
Average number of hydrogen bonds (normalized by the number of ionic pairs) in each of the NADES, determined for both the electrochemical cell and the pure electrolyte. The figure on the left shows three distinct regions where the hydrogen bonds were considered separately: in the positive and negative EDLs, as well as in the bulk region.

The electrostatic potential across the supercapacitor (or electrostatic potential profile), Φ­(z) (see Figure 6), is determined by integrating the one-dimensional Poisson equation, utilizing the local charge density derived from the atomic charge distribution of each ionic species.^32^ The local charge density is calculated based on the spatial distribution of charges and the electrode’s surface area. The supercapacitor’s capacitance (C) is influenced by the electrode’s surface charge density σ and the potential difference across the device (ΔΔΦ) (Figure 7a). Surface charge is obtained by averaging the total charge on the electrode, normalized by its surface area. The potential difference is calculated as the difference between the potential drops in charged and discharged states and then corrected by the potential of zero charge. Electrode capacitances (C ^±^ ) are derived from linear fits of σ versus ΔΔΦ and combined using the series capacitance formula to determine the total capacitance, *C*_Tot_ (Figure 7b). For all cases, charged or uncharged, Figure 6 presents the electrostatic potential profile, which exhibits typical behavior. That is, the potential varies approximately linearly in the bulk of the electrolyte due to the uniform electric field generated by the distribution of ions near the surfaces of the electrodes. At the electrode/electrolyte interfaces, abrupt changes in the electrostatic potential occur, resulting from the formation of an electric double layer, where the ions in the electrolyte reorganize in response to the charges present on the graphene surface. This distribution is essential for efficient energy storage in the device.

**Figure 6:**
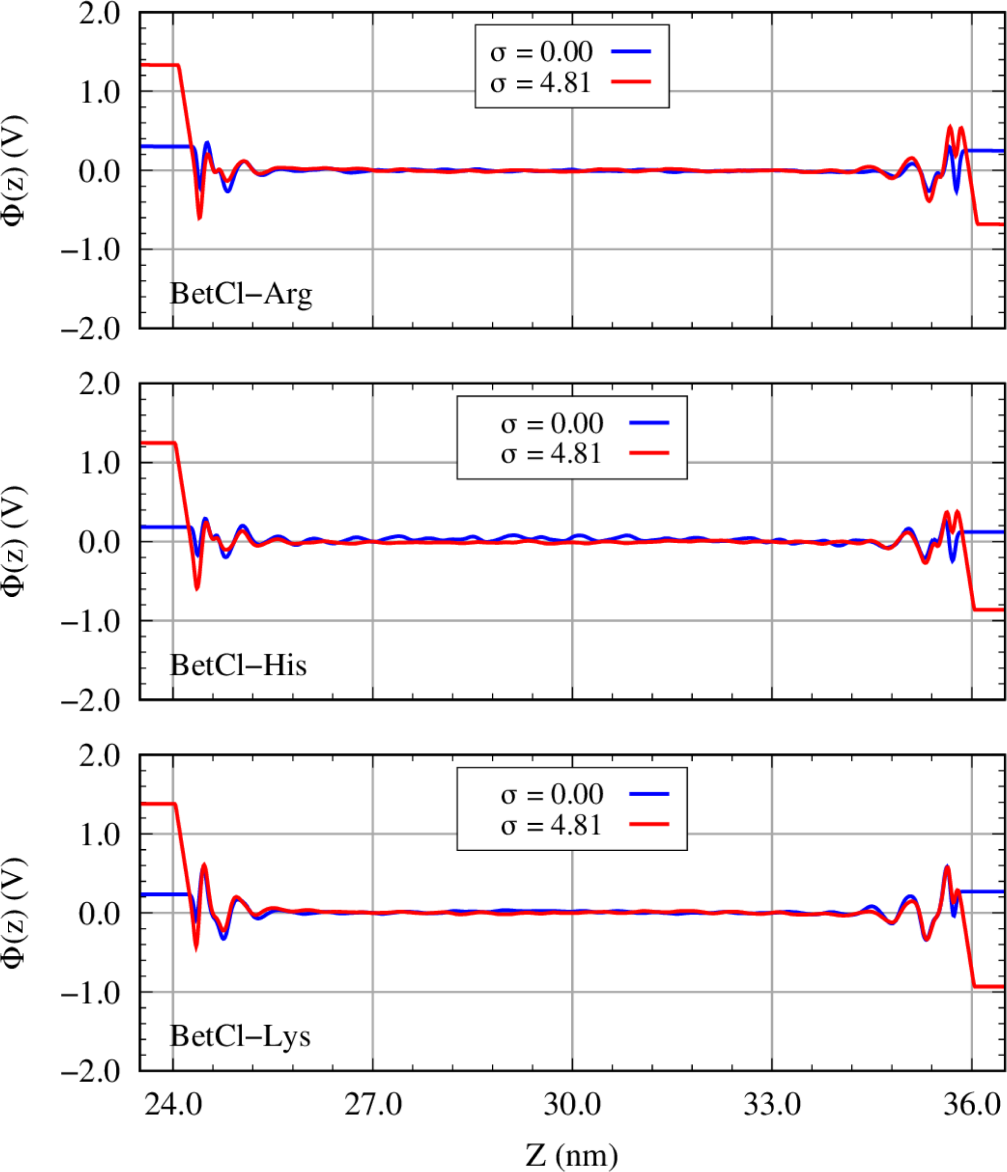
Electrostatic potential profile (in V) as a function of z distance (normal to electrode) for the three supercapacitor investigated and charge densities of 0.00 (blue line) and 4.81 μC/cm^2^ (red line).

**Figure 7:**
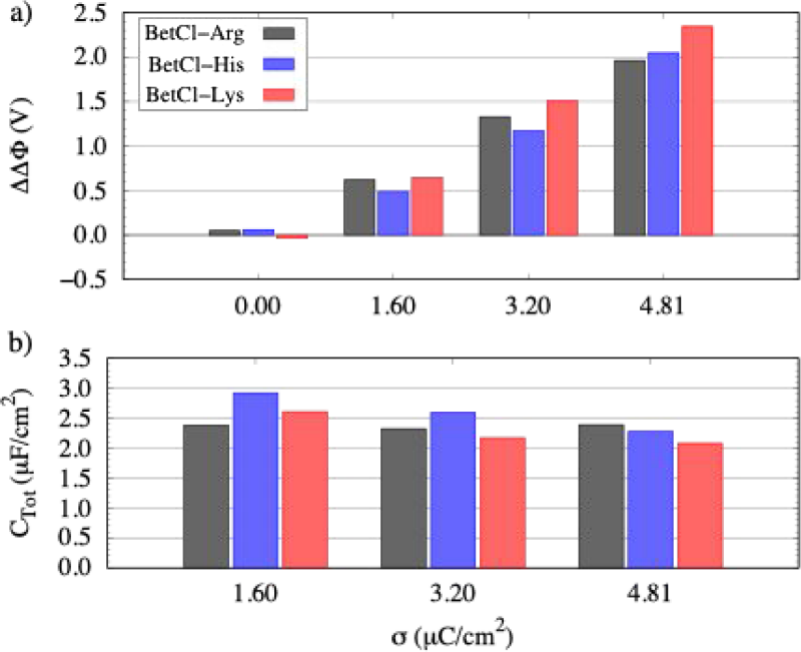
(a) At top, potential drop (ΔΔΦ, in Volts, with PZC correction); (b) at bottom, total capacitance (μF/cm^2^) for the supercapacitor models with the three electrolytes studied as a function of charge densities. The potential drop (ΔΔΦ) is corrected using the potential of zero charge (PZC), obtained from simulations at σ = 0 μC/cm^2^ by averaging the potential near the electrodes.

The potential drop across the supercapacitor (ΔΔΦ), shown in Figure 7a, is calculated as the difference between the average values of the electrostatic potential near the electrode–electrolyte interfaces of each electrode. As expected, higher surface charge promotes more pronounced layering of ionic species, particularly amino acids at the positive electrode and betaine at the negative electrode. This enhanced organization reinforces the formation of a compact EDL, directly influencing the capacitance. We observe that the potential drop varies linearly with the charge density, which is essential for understanding energy storage mechanisms and, in particular, for calculating the total capacitance. Figure 7b shows that the total capacitance value ranges between 2.2 and 2.8 μF cm^– 2^, depending on the electrolyte used in the supercapacitor and the applied voltage. In comparison with results obtained for other ionic liquids in graphene-based supercapacitors, for example, [bmim]­[PF_6_] exhibits a total capacitance *(C*_Tot_ *)* of 2.0 μF cm^– 2^,^33^ while [emim]­[BF_4_] reaches values close to *C*_Tot_ = 2.6 μF cm^– 2^,^33^ and choline- and glycine-based ionic liquids show *C*_Tot_ = 2.4 μF cm^– 2^.^33^ Furthermore, amino acid–based ionic liquids, such as those of the type [emim]­[AA], exhibit *C*_Tot_ values ranging from 2.0 to 2.7 μF cm^– 2^, depending on the amino acid used and the degree of electrolyte hydration.^31^ The histidine-based system exhibits the highest total capacitance at 1.6 μC cm^– 2^, and this value tends to decrease with voltage, a pattern that is also observed for the other electrolytes. The capacitance values exhibit magnitudes comparable to those of various other organic electrolytes and ionic liquid-based electrolytes, suggesting that NADES hold potential for exploration in energy storage applications, either as primary electrolytes or as additives capable of enhancing the properties of conventional electrolytes.

Finally, the energy stored (per total mass) in supercapacitors can be estimated using the equation*u* = *CV* ^2^/2, where C is the total capacitance of the device and V is the potential difference corresponding to each sigma value. For a target potential difference of 2 V, the system with charges rescaled to 80% composed of BetCl-Arg can store up to 3.09 J/g. The system composed of BetCl-His reaches up 3.00 J/g, and BetCl-Lys achieves approximately 2.88 J/g. The energy storage difference between NADES systems is small (∼7% for rescaled charge systems). However, a comparison with other systems highlights their performance. Devices composed of graphene and traditional ionic liquids, such as [bmim]­[PF_6_] at 2.5 V, store approximately 3.35 J/g,^33^ while [emim]­[BF_4_] at 1.8 V stores only 1.83 J/g.^33^ Organic ionic liquids based on choline and glycine at ∼ 2 V store around 2.11 J/g.^33^ Another study demonstrates that amino acid–based ionic liquids ([emim]­[AA]) exhibit gravimetric energy densities of approximately 3.11 J/g, 2.83 J/g, 3.17 J/g, and 2.96 J/g when composed of alanine, valine, leucine, and isoleucine, respectively.^31^ The authors also noted a gradual increase in these values when the ionic liquid was hydrated, with gains of 34–50% observed for hydration levels up to 90%. These findings suggest that the NADES electrolytes used in this study are highly promising compared to traditional ionic liquids ([emim]­[BF_4_] and [bmim]­[PF_6_]), performing similarly to amino acid–based ionic liquids of the [emim]­[AA] type. This indicates that the performance of these electrolytes could be further enhanced through hydration.

A comparison with previous studies reinforces the relevance of our results. In particular, Kaur et al. investigated the behavior of the eutectic solvent reline (a mixture of choline chloride and urea) at graphene electrodes through classical molecular dynamics simulations, reporting differential capacitance values ranging from 1.8 to 3.0 μF/cm^2^, depending on the surface charge density and the interfacial organization of the liquid.^34^ These values are comparable to those found in the present work for BetCl-based systems, despite differences in electrolyte composition and simulation temperature. Furthermore, both studies observed well-defined ionic layering and asymmetries in the electric double layer structure, sensitive to the surface polarization. The close agreement in capacitance values suggests that NADES-based systems can offer competitive electrochemical performance, with the added advantage of being composed of biodegradable and low-toxicity components; enhancing their potential as sustainable alternatives for energy storage devices.

## Conclusions

In this work, we investigate the use of DES as electrolytes for graphene-based supercapacitors, using classical molecular dynamics simulations. Three different NADES were considered, formed by betaine/chloride and the amino acids arginine, histidine, and lysine. It was observed that, in terms of intrinsic properties, histidine-based electrolytes exhibited the highest mass density and cohesive energy, while lysine-based systems showed the highest diffusion coefficients, confirming the sensitivity of ionic mobility to temperature. From the perspective of interactions with the electrodes, simulations in supercapacitors revealed that amino acids play a predominant role in the formation of the electric double layer on the positive electrode, while betaine contributes significantly on the negative electrode, highlighting the asymmetric nature of interactions between electrolyte components and electrodes.

The study examined the hydrogen bond (HB) network in pure electrolytes and under confinement within supercapacitor electrodes. It was found that the average HB count in the electric double layers (EDLs) is similar to that in the bulk electrolyte, resembling the behavior of amino acid–based ionic liquids with low hydration levels. Despite this uniformity, arginine-based NADES exhibited the highest average number of HBs per amino acid (2.0–2.5 in the device), followed by histidine and lysine (∼1.3). Furthermore, the positive electrode was observed to have a higher density of amino acids, with distinct compositions in the EDL regions. These findings underscore the structural organization and interactions of NADES under confinement, highlighting their influence on the electrochemical behavior of the system.

Analysis of the electrostatic potential profile and capacitance values showed that histidine-based systems achieved the highest total capacitance values, ranging from 2.2 to 2.8 μF/cm^2^, depending on the applied charge density. These results, combined with the low toxicity, biodegradability, and affordability of NADES, demonstrate their great potential to replace or complement conventional electrolytes in supercapacitors.

It is important to note, however, that NADES are still in the early stages of investigation as supercapacitor electrolytes. Their high viscosity and extensive hydrogen bonding network offer advantages such as thermal stability and safety, but also pose challenges like reduced ionic mobility. These limitations currently hinder their direct replacement in commercial devices. Nevertheless, their sustainability, low toxicity, and ease of preparation make NADES highly promising for green energy storage applications, particularly where environmental and safety concerns are paramount. While a quantitative economic evaluation is premature due to the lack of large-scale production data, the use of inexpensive and abundant components (e.g., amino acids and betaine) suggests a potentially low-cost alternative for specific applications.

Thus, this study advances the understanding of the electrochemical behavior of NADES and reinforces their applicability as a promising solution for sustainable energy storage devices. Future work could explore new NADES designs by varying the proportions of their components or investigating the influence of different electrodes to maximize the performance of these systems.

## Supplementary Material





## Notes

Diffusion coefficients and mean square displacement (MSD) plots for electrolyte components under different conditions (300 K, 600 K, charged and uncharged supercapacitors); includes comparative analysis of ionic mobility­(PDF)
Molecular structure (.pdb) and force field parameters (.itp); atomic coordinates and topology data used in molecular dynamics simulations with GROMACS­(ZIP)
