# Supplementary material for: Unveiling the Potential of Natural Deep Eutectic Solvents in Electrochemical Energy Storage Applications
Source: ACS Phys Chem Au. 2025 Aug 15;6(1):29–37. doi: 10.1021/acsphyschemau.5c00063 (PMC12856660; doi:10.1021/acsphyschemau.5c00063)
Supplement: Supplementary file 1 [file pg5c00063_si_001.pdf]

S U P P O R T   M A T E R I A L

Unveiling the Potential of Natural Deep Eutectic Solvents in Electrochemical Energy Storage Applications

Henrique de Araujo Chagas[1], Guilherme Colherinhas[1], and Eudes E. Fileti[2]*

[1] *Instituto de Física, Universidade Federal de Goiás, 74690-900, Goiânia, GO, Brazil.*

[2] *Instituto de Ciência e Tecnologia, Universidade Federal de São Paulo, 12247-014, São José dos Campos, São Paulo, Brazil.*

* *corresponding author: gcolherinhas@ufg.br (GC)*

---

* gcolherinhas@ufg.br (corresponding author)

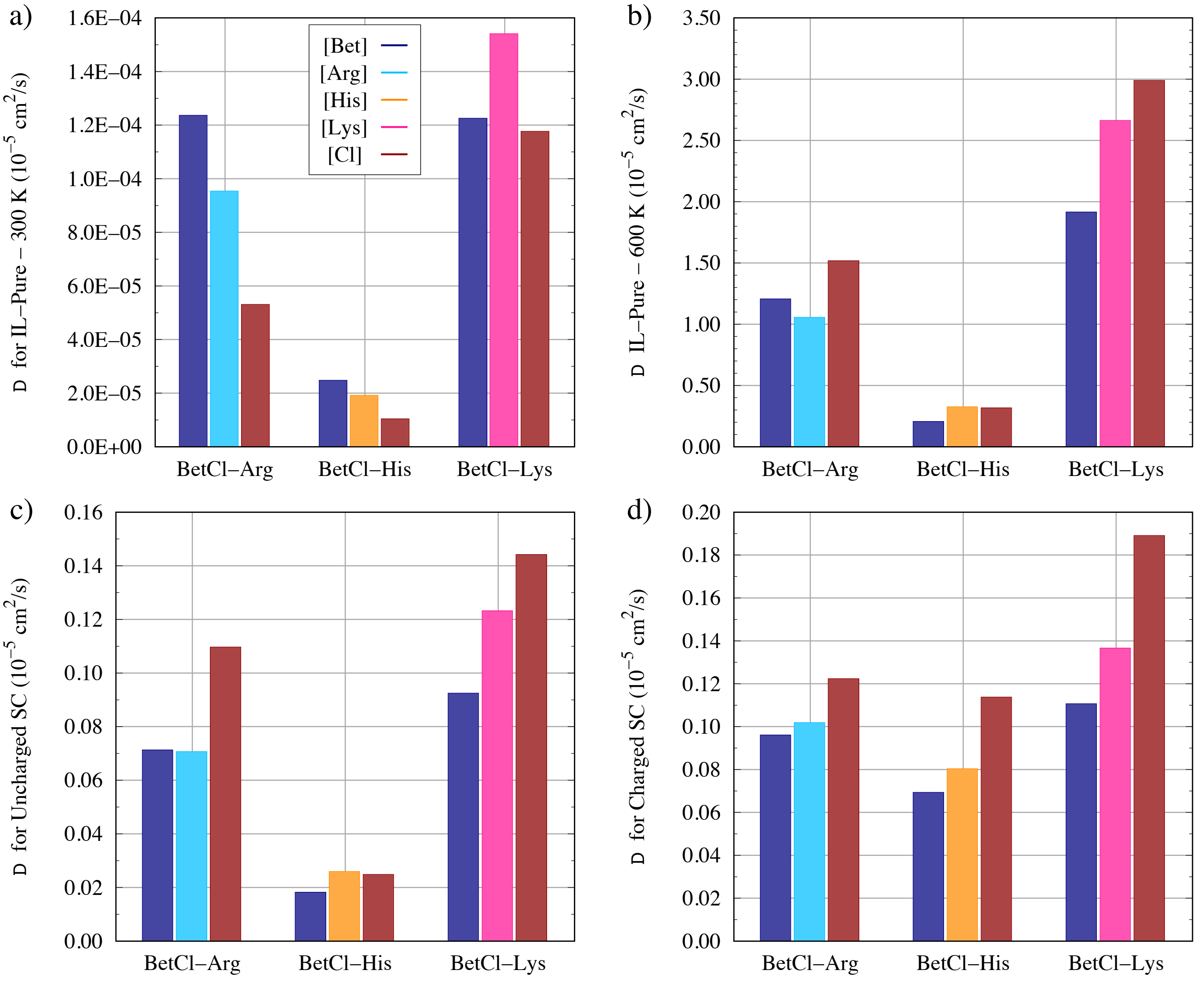

Figure S1: *Diffusion coefficient (D) (in cm^2/s) for each component of the electrolytes: a) IL-Pure at 300 K, b) IL-Pure at 600 K, c) Uncharged supercapacitor at 600 K, and d) Charged supercapacitor at 600 K.*

Table S1: *Diffusion coefficient (D) (in 10^{-5} cm^2/s) of electrolyte components for the studied systems: IL-Pure at 300 K and 600 K, and uncharged and charged supercapacitors.*

Electrolyte		***IL-Pure***		***Supercapacitor***	
		300 K	***600 K***	***Uncharged***	***Charged***
BetCl-Arg	***[Bet]***	*0.0001237*	*1.2074*	*0.0713*	*0.0961*
	[Arg]	*0.0000954*	*1.0571*	*0.0707*	*0.1019*
	[Cl]	*0.0000531*	*1.5191*	*0.1097*	*0.1224*
BetCl-His	***[Bet]***	*0.0000248*	*0.2068*	*0.0182*	*0.0694*
	[His]	*0.0000192*	*0.3261*	*0.0260*	*0.0804*
	[Cl]	*0.0000104*	*0.3178*	*0.0249*	*0.1138*
BetCl-Lys	***[Bet]***	*0.0001226*	*1.9160*	*0.0925*	*0.1107*
	[Lys]	*0.0001541*	*2.6634*	*0.1232*	*0.1366*
	[Cl]	*0.0001177*	*2.9904*	*0.1442*	*0.1891*

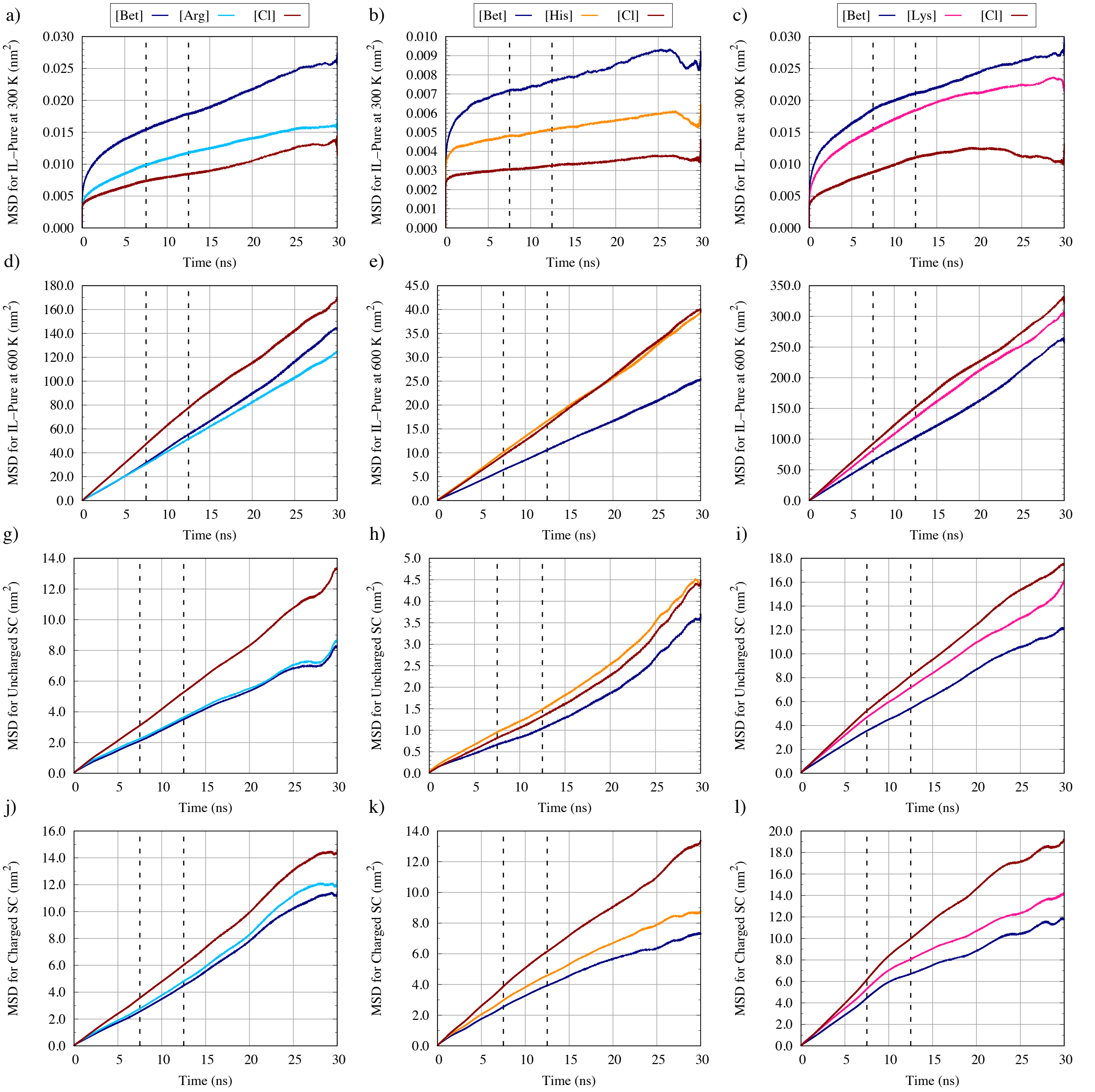

Figure S2: *Mean Square Displacement (MSD) (in* nm^2*) for each component of the electrolytes: a) IL-Pure at 300 k, b) IL-Pure at 600 K, c) Uncharged supercapacitor and d) Charged supercapacitor. The dashed lines indicates the regions used to calculate the diffusion coefficients.*
